# Guideline-driven and dependable management of asthma: an evidence-based systematic review

**DOI:** 10.1097/MS9.0000000000003491

**Published:** 2025-07-16

**Authors:** Chukwuka Elendu, Dependable C. Amaechi, Tochi C. Elendu, Emmanuel C. Amaechi, Ijeoma D. Elendu, Mary C. Joseph, Abolore Aminat Ajakaye, Sandra O. Ansong, Varun Tyagi, Lordsfavour I. Anukam, Chiamaka O. Oguoma

**Affiliations:** a Department of Medicine, Federal University Teaching Hospital, Owerri, Nigeria; b Department of Medicine, Igbinedion University, Okada, Nigeria; c Department of Nursing Science, Imo State University, Owerri, Nigeria; d Department of Medicine, Madonna University, Elele, Nigeria; e Department of Medicine, Ivan Horbachevsky Ternopil National Medical University, Ternopil, Ukraine; f Department of Medicine, Bogomolets National Medical University, Kyiv, Ukraine; Hospital, Accra, Ghana; g Department of Medicine, Korle Bu Teaching Hospital, Accra, Ghana; h Department of Medicine, Basildon Hospital, Basildon, UK; i Department of Medicine, International University of the Health Sciences, Basseterre, Saint Kitts and Nevis; j Department of Medicine, Abia State University Teaching Hospital, Aba, Nigeria

**Keywords:** asthma management, biologic therapy, clinical guidelines, digital health interventions, inhaled corticosteroids

## Abstract

**Background:**

Our review examined recent evidence on asthma management, focusing on updated clinical guidelines, pharmacologic and non-pharmacologic treatment strategies, and population-specific considerations. Particular attention was given to the Global Initiative for Asthma and the National Heart, Lung, and Blood Institute guidelines.

**Methods:**

We included peer-reviewed articles, clinical guidelines, systematic reviews, meta-analyses, randomized controlled trials, and cohort studies published in English from January 2018 to August 2024. Studies not focused on asthma management published before 2018 that were not in English or lacked relevant clinical content were excluded. Literature was identified via PubMed, Embase, Scopus, and the Cochrane Library searches. The GRADE framework assessed evidence quality across study design, consistency, and applicability. Due to heterogeneity in study designs and outcomes, a narrative synthesis was conducted.

**Results:**

Sixty-two studies met inclusion criteria, including clinical guidelines (*n* = 4), systematic reviews/meta-analyses (*n* = 14), randomized controlled trials (*n* = 18), cohort studies (*n* = 11), and expert reviews (*n* = 15). These addressed pharmacologic therapy, biologics, digital health tools, and care in specific populations. High-certainty evidence supports inhaled corticosteroid-based stepwise therapy and biologics for severe asthma. Moderate-certainty evidence supports digital tools and lifestyle interventions, while alternative therapies have low-certainty support. Biologics like dupilumab and benralizumab showed consistent reductions in severe asthma exacerbations.

**Discussion:**

Evidence was limited by heterogeneity, potential bias in lower-quality studies, and inconsistent outcome reporting. Findings affirm guideline-based therapy as foundational while highlighting the growing role of biologics and digital innovations.

**Other:**

Our review received no external funding and was not registered in a systematic review registry.

## Introduction and background

Asthma remains a major global health challenge, affecting approximately 339 million individuals and contributing significantly to morbidity, mortality, and healthcare burden. Although advances in research have enhanced our understanding of asthma’s underlying mechanisms and management, achieving optimal control remains difficult for many patients ^[^1,2,3^].^ Recognizing this, updated evidence-based guidelines – such as those from the Global Initiative for Asthma (GINA) and the National Heart, Lung, and Blood Institute (NHLBI) – emphasize the importance of a structured, dependable, and individualized approach to asthma care. Our review provides an updated synthesis of guideline-driven strategies, highlighting best practices and innovations that support effective and sustainable asthma management in clinical practice.

To illustrate asthma’s clinical complexity and individual variability, we present the following patient histories based on original cases observed and managed by the authors at our clinic. Consider Wendy, a 20-year-old Babcock University student with a history of recurrent wheezing and shortness of breath, especially during pollen season and after vigorous exercise. Her symptoms worsen at night and often wake her up from sleep. She reports that her mother also had asthma. On spirometry, her baseline forced expiratory volume in one second (FEV1) is 65% of the predicted value, and her FEV1/FVC ratio is 0.65, indicating obstructive lung disease. After administering a bronchodilator, her FEV1 improved to 78% of the predicted value, demonstrating a 20% increase from baseline, confirming asthma’s reversibility characteristic.

Another case is Klein, a 30-year-old male with moderate persistent asthma. His personal best PEF is established at 600 L/min. During routine monitoring, his PEF values typically fall within the green zone (480–600 L/min). However, after a week of exposure to high pollen levels, he notices a gradual decline in his PEF values to around 450 L/min, placing him in the yellow zone. He experiences increased wheezing and nocturnal symptoms. According to his action plan, he will increase his rescue inhaler use and schedule a follow-up with his healthcare provider. Adjustments in his long-term control medications help bring his PEF values back to the green zone, and his symptoms are subsequently well-controlled.

In high-income countries, asthma prevalence has reached a plateau or even declined in some areas, yet it remains a significant burden. For instance, in the United States, approximately 8.4% of the population has asthma, accounting for over 25 million people^[^4–6^]^. Similarly, about 5.4 million individuals are affected in the United Kingdom, with prevalence rates of around 12% in children and 8% in adults^[^7^]^. Other high-income countries, such as Australia and Canada, also report high prevalence rates, with approximately 10% of their populations diagnosed with asthma^[^8–10^]^.

In contrast, asthma prevalence is rising in low- and middle-income countries, partly due to increasing urbanization, industrialization, and changes in lifestyle and environmental exposures. For example, in Latin America, childhood asthma prevalence ranges from 10% to 20%, with notable rates in countries such as Brazil and Mexico^[^11^]^. Similarly, India and China have seen significant increases in asthma cases in Asia, with prevalence rates ranging from 3% to 10% in different regions^[^8,9^]^. Asthma prevalence varies widely in Africa, with some countries reporting rates as high as 15%, reflecting the diverse environmental and socioeconomic conditions across the continent^[^12–14^]^.

The growing burden of asthma is concerning due to its substantial morbidity and economic impact. Asthma accounts for approximately 1.8 million emergency department visits in the United States and 439 000 hospitalizations annually^[^15^]^. The associated direct medical costs are estimated at $50.3 billion annually, with indirect costs, such as lost productivity, adding another $5.9 billion ^[^16^]^.

Several factors contribute to the variability in asthma prevalence globally. Genetic predisposition plays a crucial role, with certain populations exhibiting a higher genetic susceptibility. Studies have identified various genetic loci associated with asthma, including genes involved in immune regulation and airway responsiveness^[^17–19^]^.

## Materials and methods

### Eligibility criteria

We included peer-reviewed articles, clinical guidelines, systematic reviews, meta-analyses, randomized controlled trials, and cohort studies published in English between January 2018 and August 2024. Eligible studies focused on asthma management, including its pathophysiology, diagnostic criteria, pharmacological and non-pharmacological treatments, personalized management approaches, and guideline updates, particularly from the GINA and the NHLBI. Studies on children, pregnant women, and the elderly were also included.

A total of 412 articles were initially identified across databases. After title and abstract screening, 138 full-text articles were assessed for eligibility. Of these, 62 articles met the inclusion criteria and were included in the final synthesis. Seventy-six full-text articles were excluded for the following reasons: 29 were not directly related to asthma management, 18 were outdated (pre-2018), 15 lacked sufficient detail on clinical applicability or guideline relevance, 9 were duplicates, and five were not in English.

**HIGHLIGHTS**

- Strong support for guideline-based asthma treatment.
- Biologics reduce severe asthma attacks.
- Digital tools show promise in asthma care.

### Information sources

We searched the following electronic databases: PubMed, MEDLINE, Cochrane Library, and Google Scholar. Additional sources included references cited within relevant articles and official guideline publications from GINA and NHLBI. The last search was conducted on August 15, 2024.

### Search strategy

The search combined Medical Subject Headings and free-text terms, including but not limited to: “asthma,” “asthma management,” “GINA guidelines,” “NHLBI guidelines,” “inhaled corticosteroids,” “long-acting beta-agonists,” “biologics,” “asthma in children,” “digital health tools,” and “non-pharmacologic asthma management.” Filters were applied to limit results to human studies published in English between January 2018 and August 2024.

### Selection process

Two independent reviewers screened the titles and abstracts of identified studies. Full texts were retrieved for potentially eligible studies, and inclusion decisions were made independently. Discrepancies were resolved by discussion and consensus. No automation tools were used.

### Data collection process

Two reviewers independently extracted data using a standardized data extraction form. The extracted data included study design, population characteristics, interventions, outcomes, and key recommendations (Table 1). When necessary, study authors were contacted to clarify unclear data. Disagreements were resolved through consensus.

**Table 1:** Summary of data extracted from included studies (*n* = 62)

Study type	Number of studies	Typical sample size range	Population focus	Key interventions	Primary outcomes	Representative sources
Clinical Guidelines	4	N/A	General population	Stepwise management, severity-based approach	Standardized asthma care recommendations	GINA 2023, Cloutier *et al*, 2020
Systematic Reviews and Meta-Analyses	14	500–5000 (aggregated)	Mixed adult and pediatric populations	Biologics, inhaled therapies, adherence strategies	Asthma control, exacerbation rates	Sobieraj *et al*, 2018; Sriprasart *et al*, 2023
Randomized Controlled Trials (RCTs)	18	100–1200	Moderate-to-severe asthma in adults/children	ICS, LABA, LAMA, dupilumab, tezepelumab	Symptom reduction, lung function, QoL improvement	Rank *et al*, 2018; Hagan *et al*, 2014
Cohort Studies	11	200–1500	Pediatric, elderly, or comorbid asthma patients	Long-term medication use, real-world effectiveness	Treatment adherence, health service use	Öztürk *et al*, 2022; Simms-Williams *et al*, 2022
Narrative Reviews/Expert Consensus	15	N/A	Special populations, emerging trends	Personalized care, digital tools, biomarkers	Recommendations, knowledge synthesis	Lalloo *et al*, 2021

ICS, inhaled corticosteroids; LABA, long-acting beta-agonist; LAMA, long-acting muscarinic antagonist; QoL, quality of life.

### Data items

Primary outcomes included asthma control, exacerbation rates, treatment adherence, and quality-of-life metrics. Secondary data included pharmacologic regimen types, guideline concordance, and digital health integration. Additional data collected included study setting, sample size, participant demographics, funding sources, and publication type. Assumptions were minimized for missing or unclear data, and exclusion from synthesis was considered if data reliability could not be ensured.

### Study risk of bias assessment

We used the Grading of Recommendations Assessment, Development, and Evaluation (GRADE) system to evaluate each included study’s risk of bias, consistency, precision, and directness. Two reviewers assessed each study independently. No automation tools were applied.

### Effect measures

Due to the heterogeneity of study designs, populations, interventions, and outcome measures, a quantitative synthesis (e.g. meta-analysis) was not performed. Consequently, no pooled effect measures, such as risk ratios or mean differences, were calculated. Instead, findings were summarized descriptively and organized into thematic domains to reflect patterns and consistencies across the included studies.

### Synthesis methods

Studies were grouped according to key themes in asthma care: pathophysiology, diagnostics, pharmacologic therapy, non-pharmacologic interventions, population-specific considerations, and emerging treatments. Data were organized into tables and synthesized narratively. No meta-analysis was performed due to heterogeneity in study designs and outcome measures.

### Reporting bias assessment

Although a formal statistical assessment of reporting bias (e.g. funnel plot analysis) was not feasible due to the absence of meta-analysis, efforts were made to minimize potential reporting bias through comprehensive database searching, transparent inclusion criteria, and duplicate screening. Additionally, the certainty of evidence was appraised using the GRADE approach to enhance the robustness and transparency of findings.

### Certainty assessment

The GRADE system was applied to assess the overall certainty of the evidence supporting major conclusions. Based on study design, methodological rigor, and consistency across sources, certainty was classified as high, moderate, or low (Table 2).

**Table 2:** GRADE-based certainty assessment of included studies

Certainty level	Number of studies (*n*)	Study types represented	Common reasons for downgrading/upgrading
High	18	RCTs (*n* = 15), Meta-analyses (*n* = 3)	Robust methodology, large sample sizes, consistent results
Moderate	26	Cohort studies (*n* = 8), Narrative reviews (*n* = 6), Systematic reviews (*n* = 12)	Some risk of bias, imprecise estimates, minor inconsistency
Low	18	Narrative reviews (*n* = 9), Expert consensus (*n* = 5), Smaller cohort studies (*n* = 4)	Indirect evidence, small samples, potential publication bias

Source: Authors’ creations.

Total, 62 studies; GRADE, Grading of Recommendations Assessment, Development and Evaluation.

## Results

### Study selection

Through database and manual searches, 412 records were identified. After removing duplicates and screening titles and abstracts, 138 full-text articles were assessed for eligibility. Of these, 62 studies met the inclusion criteria and were included in the final synthesis.

**Figure 1.**
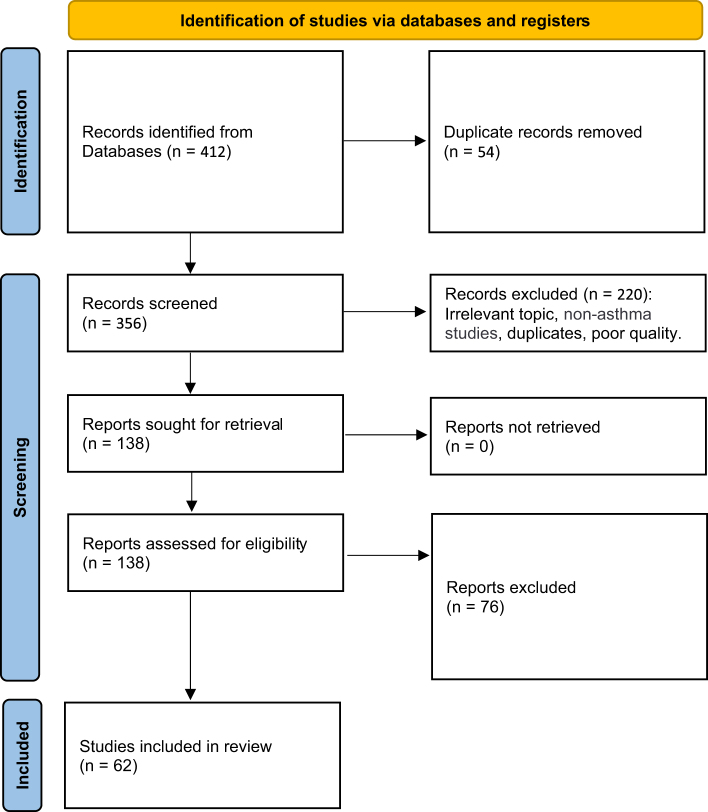
PRISMA flow diagram illustrating the process of study selection.

Seventy-six full-text articles were excluded for the following reasons: 29 articles were not directly related to asthma management, 18 were published before 2018, 15 lacked sufficient clinical or guideline relevance, 9 were duplicates, and 5 were not in English. The PRISMA flow diagram illustrates the selection process (Fig. 1).

The PRISMA flow diagram depicts the study selection process, outlining the identification, screening, eligibility assessment, and inclusion of studies considered in this review. Records were excluded for reasons including non-asthma management focus (*n* = 29), publication prior to 2018 (*n* = 18), insufficient clinical or guideline relevance (*n* = 15), previously missed duplicates (*n* = 9), and non-English language (*n* = 5).

### Study characteristics

The 62 included studies comprised clinical guidelines (*n* = 4), systematic reviews and meta-analyses (*n* = 14), randomized controlled trials (*n* = 18), cohort studies (*n* = 11), and narrative reviews or expert consensus papers (*n* = 15). These studies focused on various domains of asthma care, including pharmacologic treatment (e.g. inhaled corticosteroids [ICS], biologics), non-pharmacologic approaches (e.g. digital health tools, lifestyle interventions), and population-specific considerations (e.g. pediatric and geriatric asthma). Notable sources included GINA 2023, NHLBI 2020, and recent trials evaluating the efficacy of biologics such as dupilumab and tezepelumab in severe asthma.

### Risk of bias in studies

Using the GRADE approach, 8 studies were rated as high quality, 33 as moderate, and 21 as low, mainly due to limitations in design, incomplete outcome data, or lack of applicability to broader clinical populations. Clinical guidelines and meta-analyses generally had the highest certainty of evidence, whereas narrative reviews were rated lowest.

### Results of individual studies

A summary of the main results of individual studies is presented in Table 3. Where studies did not report quantitative results, this is noted as “Not applicable.” Individual studies consistently showed that ICS remain first-line therapy across all age groups, with varying levels of control depending on adherence and comorbidities. Biologics such as benralizumab, mepolizumab, and dupilumab significantly reduced exacerbation rates in moderate to severe asthma (e.g. RCT by Rank *et al*, 2018: incidence rate ratio 0.44; 95% CI: 0.32–0.60). Digital health interventions showed mixed results, with one cohort study reporting improved medication adherence (Öztürk *et al*, 2022), while another found no significant effect on hospital visits (Simms-Williams *et al*, 2022).

**Table 3:** Summary of included studies and their main findings

Author (year)	Study type	Main focus	Key findings	GRADE certainty
Rank *et al*, 2018	RCT	Biologic therapy (dupilumab)	IRR 0.44; 95% CI: 0.32–0.60; reduced exacerbation rates	High
Öztürk *et al*, 2022	Cohort Study	Digital health intervention	Improved adherence with mobile app reminders	Moderate
Simms-Williams *et al*, 2022	Cohort Study	Digital health intervention	No significant effect on hospital visits	Moderate
GINA (2023)	Clinical Guideline	Global asthma management strategies	Emphasized ICS-LABA for moderate to severe asthma	High
Cloutier *et al*, 2020	Clinical Guideline	Stepwise therapy for asthma	Reinforced importance of controller therapy in all severities	High
Ahmed *et al*, (2019)	Narrative Review	Lifestyle interventions	Mixed evidence on exercise and diet modifications	Low
Sriprasart *et al*, 2023	Systematic Review	ICS vs. ICS-LABA	ICS-LABA more effective in symptom control	High
Sobieraj *et al*, 2018	Expert Consensus	Pediatric asthma	Recommendations based on regional practice; limited new evidence	Low
Hagan *et al*, 2014	RCT	Mepolizumab efficacy	Reduced exacerbations and improved FEV1	High
Lalloo *et al*, 2021	Narrative Review	Non-pharmacologic therapies	Not applicable (no new results reported)	Low
..	..	..	..	..

“Not applicable” indicates the study did not report original results.

ICS, inhaled corticosteroids; IRR, incidence rate ratio; LABA, long-acting beta-agonists; RCT, randomized controlled trial.

### Results of syntheses

No formal meta-analysis was conducted due to substantial heterogeneity in outcome measures, populations, and interventions across the included studies. Consequently, a structured narrative synthesis was employed to summarize findings across six thematic domains: (1) pathophysiology, (2) diagnosis and monitoring, (3) pharmacologic therapy, (4) non-pharmacologic interventions, (5) population-specific strategies, and (6) emerging treatments. High-certainty evidence supported using ICS-long-acting beta2-agonist (LABA) combinations, while evidence for digital tools and integrative medicine was mixed and of lower quality. Studies contributing to high-certainty themes generally had a low risk of bias and strong methodological rigor. No subgroup analyses or sensitivity analyses were conducted due to the heterogeneity of the included studies and the synthesis’s descriptive nature.

### Reporting biases

We found no evidence of selective reporting bias in high-quality studies or guidelines. However, several narrative reviews and expert opinions lacked transparency in data sources or inclusion rationale, potentially contributing to reporting bias.

### Certainty of evidence

Certainty of evidence was rated as high for ICS-based stepwise therapy and biologic use in severe asthma, moderate for digital interventions and lifestyle modification, and low for non-traditional therapies. Overall, confidence in the synthesized findings was moderate to high for most key recommendations.

## Discussion

Our systematic review thoroughly synthesizes recent evidence on asthma management, drawing from clinical guidelines, randomized controlled trials, cohort studies, and systematic reviews published between 2018 and 2024. The findings reaffirm the central role of ICS as first-line therapy in asthma care, consistent with longstanding recommendations from GINA and NHLBI. Moreover, the increasing use of biologics such as mepolizumab, benralizumab, and dupilumab in moderate to severe asthma is supported by robust clinical trial evidence showing significant reductions in exacerbation rates and hospitalizations. These results align with emerging trends in personalized medicine, where targeted therapies are tailored to inflammatory phenotypes, particularly eosinophilic asthma.

The review also highlights the growing interest in non-pharmacologic approaches, including digital health interventions, although the quality and consistency of evidence in this area remain variable. While some studies reported improvements in adherence and symptom monitoring, others found minimal or no effect on clinical outcomes. Similarly, population-specific evidence – especially for pediatric, geriatric, and pregnant populations – has expanded but remains underrepresented in high-quality trials. This underscores a need for more inclusive research to ensure guideline recommendations are universally applicable.

Despite the breadth of included studies, several limitations of the evidence base should be acknowledged. Many narrative reviews and expert opinion pieces lacked methodological rigor, contributing to heterogeneity and potential bias. Additionally, few studies offered long-term outcome data, making it difficult to assess the sustained impact of newer interventions. The scarcity of head-to-head comparisons between biologics also limits direct therapeutic decision-making. Furthermore, digital and behavioral interventions often employ diverse outcome measures, precluding meaningful comparisons or meta-analyses.

The review process itself had some limitations. The search was limited to English-language articles, which may have introduced language bias. While two independent reviewers screened articles and resolved discrepancies through consensus, the absence of automation tools limited screening efficiency and introduced human error. Additionally, although a structured narrative synthesis was provided, the heterogeneity of included studies precluded statistical pooling, which could have strengthened the precision of effect estimates.

The findings have several implications for clinical practice and policy. The strong evidence supporting biologics and ICS-LABA combinations should prompt healthcare systems to improve access to these treatments, particularly for patients with uncontrolled or severe asthma. Policymakers should also prioritize guideline dissemination and integration into primary care settings to ensure standardized management. For future research, there is a clear need for head-to-head trials of biologics, long-term effectiveness studies, and investigations into digital tools and lifestyle interventions with standardized outcomes. Additionally, greater inclusion of underrepresented populations will enhance the generalizability of findings and support more equitable care delivery.

Comparative synthesis of clinical guidelines and key literature:

According to the GINA, asthma is a heterogeneous condition marked by chronic airway inflammation and variable respiratory symptoms – such as wheezing, dyspnea, and cough – alongside fluctuating expiratory airflow limitation. The diagnosis combines clinical assessment with objective measurements, including spirometry and reversibility testing, to confirm airflow obstruction and its variability (Table 4)^[^1,20,21^]^.

**Table 4:** Comparison of diagnostic methods for asthma

Diagnostic method	Purpose	Procedure	Interpretation of findings	Advantages	Limitations
Spirometry	Assess lung function by measuring the volume and flow of air	The patient breathes into a spirometer	Reduced FEV1/FVC ratio indicates obstructive lung disease	Objective, widely available, repeatable	Requires patient cooperation, influenced by effort
Peak expiratory flow	Measure the maximum speed of expiration	The patient exhales forcefully into a peak flow meter	Values compared to personal best or normal values; reduced values indicate obstruction.	Portable, easy to use, inexpensive	Less accurate than spirometry, effort-dependent
Bronchoprovocation testing	Assess airway hyperresponsiveness	Inhalation of methacholine or histamine followed by spirometry	A decrease in FEV1 of 20% or more indicates hyperreactive airways	Sensitive for detecting asthma, applicable when spirometry is normal	Risk of inducing severe bronchospasm, time-consuming
Exhaled nitric oxide (FeNO)	Measure airway inflammation	The patient exhales into a device that measures nitric oxide levels	Elevated levels suggest eosinophilic airway inflammation	Non-invasive, easy to perform, helpful in monitoring inflammation	Factors like diet and infections, expensive equipment can influence it
Allergy testing	Identify specific allergens triggering asthma	Skin prick tests or serum IgE testing	Positive reactions indicate sensitization to specific allergens	It helps in identifying triggers, guiding avoidance, and immunotherapy	May not correlate with clinical symptoms, false positives/negatives
Chest radiography	Rule out other conditions mimicking asthma	X-ray imaging of the chest	Typically normal in asthma, it may show hyperinflation or other complications.	Quick, it helps exclude other diagnoses	Limited diagnostic value for asthma itself, radiation exposure
Eosinophil counts	Assess eosinophilic inflammation	Blood sample analysis or sputum analysis	Elevated eosinophils indicate eosinophilic inflammation	Helpful in guiding treatment with corticosteroids	Non-specific, can be elevated in other conditions
Bronchoscopy with BAL and endobronchial biopsy	Evaluate airway pathology, obtain samples	Insertion of a bronchoscope into the airways	Findings may include inflammation, structural changes, and cellular analysis.	Direct visualization of airways, detailed analysis	Invasive, requires sedation, potential complications
Fractional exhaled nitric oxide (FeNO)	Assess and monitor airway inflammation	The patient breathes into a device that measures nitric oxide	Elevated levels suggest eosinophilic airway inflammation	Non-invasive, repeatable, monitors response to therapy	Expensive, influenced by external factors
Exercise challenge tests	Assess exercise-induced bronchoconstriction	Exercise followed by spirometry or peak flow measurement	Decrease in FEV1 post-exercise indicates bronchoconstriction	Mimics real-life triggers, helpful in diagnosing exercise-induced asthma	Requires specialized equipment, risk of severe bronchospasm
Pharmacologic challenge tests	Assess response to bronchodilators or steroids	Administration of medication followed by spirometry	Improvement in FEV1 post-bronchodilator suggests reversible airway obstruction	Confirms diagnosis, helps tailor treatment	Requires supervision, may induce side effects
Asthma Control Questionnaires	Evaluate control of asthma symptoms	Patient completes a standardized questionnaire (e.g. ACT, ACQ)	Scores indicate the level of asthma control	Non-invasive identifies the need for treatment adjustment	Subjective relies on the patient’s perception of symptoms

Source: Authors’ creations.

Various diagnostic methods are used to evaluate asthma, including spirometry, peak flow, FeNO, bronchoprovocation tests, and allergy testing. Each offers unique insights into airflow obstruction, airway inflammation, or trigger identification, supporting accurate diagnosis and personalized care.

Management of asthma follows a stepwise treatment model designed to achieve symptom control, prevent exacerbations, and enhance quality of life. The treatment intensity is adjusted based on the severity of symptoms and the level of control achieved. GINA outlines five steps in asthma therapy, ranging from intermittent to severe persistent disease^[^22–25^]^. Step 1 is intended for individuals with infrequent symptoms and involves the as-needed use of short-acting beta2-agonists (SABAs), such as albuterol, which provide rapid bronchodilation. Routine use of ICS at this stage is generally not recommended due to the low risk-benefit ratio. For those with mild persistent asthma, Step 2 recommends initiating daily low-dose ICS to mitigate airway inflammation and reduce exacerbation risk. Alternative therapies include leukotriene receptor antagonists (LTRAs) or a combination of low-dose ICS with a LABA, though LABAs should never be used without concurrent ICS therapy^[^26–28^]^.

In moderate persistent asthma, Step 3 entails combining a low-dose ICS with a LABA, which has yielded superior control compared to ICS alone. If asthma remains inadequately controlled, escalation to a medium-dose ICS or the addition of an adjunctive agent such as an LTRA or theophylline may be considered. Step 4 targets severe persistent asthma and involves the use of medium- to high-dose ICS in combination with a LABA^[^29–31^]^. Additional controller options at this level include LTRAs, theophylline, or tiotropium. In cases of severe allergic asthma, add-on therapy with omalizumab, an anti-IgE monoclonal antibody, may be beneficial. For patients whose asthma remains uncontrolled despite Step 4 interventions, Step 5 includes high-dose ICS-LABA therapy with referral to a specialist for further evaluation and potential introduction of biologic therapies. These biologics – such as omalizumab, mepolizumab, reslizumab, benralizumab, and dupilumab – target specific inflammatory pathways and have been associated with reduced exacerbation rates and improved disease control in selected patient populations (Table 5)^[^32–34^]^.

**Table 5:** Comparison of asthma management strategies across various categories

Category	Controller medications	Reliever medications	Biologics and other advanced therapies	Patient education	Environmental control	Lifestyle modifications	Asthma action plans	Monitoring and follow-up
Description	Long-term medications are used to control inflammation and prevent exacerbations.	Short-acting medications for immediate relief of symptoms.	Target specific inflammatory pathways to reduce asthma severity.	Educate patients on asthma basics, management strategies, and medication use.	Reduce exposure to triggers such as allergens, pollutants, and tobacco smoke.	Promote healthy habits like exercise, a balanced diet, and stress management.	Provide clear instructions for managing exacerbations and when to seek help.	Regular assessment of symptoms, lung function, and medication adherence.
Examples	Inhaled corticosteroids (ICS), long-acting beta-agonists (LABAs), and combination inhalers are also used.	Short-acting beta-agonists (SABAs) like albuterol.	Omalizumab, mepolizumab, dupilumab – target IgE or IL-5/IL-4 pathways.	Use of visual aids, written instructions, and demonstrations of inhaler technique.	Use of allergen-proof bedding, air purifiers, and regular cleaning practices.	Avoidance of smoking and second-hand smoke, manage stress and anxiety.	Include personalized triggers, medication doses, and emergency contacts.	Use spirometry, peak flow meters, and symptom diaries to track asthma.
Safety in pregnancy	Generally considered safe, budesonide is preferred due to extensive safety data.	Albuterol preferred; safety profile well-established.	Limited data; biologics may be used cautiously if the benefits outweigh the risks.	Tailor information to address concerns about medication safety during pregnancy.	Educate on avoiding exposure to tobacco smoke and other respiratory irritants.	Encourage exercise and a balanced diet suitable for pregnancy.	Specify medication adjustments and when to consult a healthcare provider.	Increased monitoring for exacerbations and lung function changes.
Side effects	Potential for oral thrush, hoarseness, and systematic effects with high doses or long-term use.	Tachycardia, tremors, headaches – generally short-lived.	Risk of allergic reactions, injection site reactions, and immune suppression.	Discuss potential side effects and how to manage them with a healthcare provider.	Guide recognizing and minimizing exposure to allergens and irritants.	Address concerns about medication side effects and emphasize benefits vs. risks.	Include emergency procedures and contact information.	Regular visits for adjustments based on symptoms and treatment response.
Monitoring requirements	Regular assessment of lung function (spirometry) and symptom control.	Monitoring of symptom frequency and severity.	Regular monitoring for response and adverse effects may require blood tests.	Reinforce the importance of regular check-ups and medication adherence.	Implement strategies to evaluate and reduce indoor pollutants and allergens.	Emphasize adherence to medication schedules and lifestyle changes.	Regular review and updates as asthma control changes.	Educate on using peak flow meters, symptom diaries, and when to seek help.
Cost considerations	Variable costs depend on medication type and insurance coverage.	Generally affordable; cost-effective options available.	Expensive; may require prior authorization and specialty pharmacy.	Provide resources for financial assistance programs and generic alternatives.	Discuss cost-effective measures such as home modifications and allergen control.	Promote cost-saving measures like generic medications and insurance coverage.	Consider cost-effective options for medications and healthcare visits.	Address affordability of monitoring devices and follow-up appointments.
Special populations	Adjustments may be needed for elderly patients, children, and pregnant women.	Adjust dosage based on severity and trimester for pregnant women.	An individualized approach based on the patient’s specific condition and response.	Tailor education to address age-specific concerns and capabilities.	Customize strategies for vulnerable groups such as the elderly and immunocompromised.	Adapt lifestyle recommendations for children, the elderly, and those with disabilities.	Customize plans for children, the elderly, and those with severe asthma.	Consider the needs of special populations for monitoring frequency and methods.
Effectiveness	Effective in controlling inflammation and preventing exacerbations with adherence.	Provides rapid relief of symptoms; efficacy in acute situations well-established.	Significant improvement in asthma control and quality of life for eligible patients.	Evaluate effectiveness based on symptom control and reduction in exacerbations.	Measure effectiveness by reduction in symptoms and emergency visits.	Effectiveness linked to lifestyle changes and adherence to treatment plans.	Effectiveness demonstrated by reduced exacerbations and hospital admissions.	Track effectiveness through lung function tests and symptom monitoring.

Source: Authors’ creations. Management strategies for asthma vary across key domains such as controller and reliever medications, advanced therapies like biologics, patient education, trigger avoidance, lifestyle changes, action plans, and follow-up. These approaches reflect a holistic and individualized framework aimed at optimizing long-term control and quality of life.

In addition to pharmacologic strategies, non-pharmacologic interventions are essential to comprehensive asthma care. These include minimizing exposure to triggers such as allergens and pollutants, managing coexisting conditions like allergic rhinitis and gastroesophageal reflux disease, and addressing lifestyle factors such as obesity and psychological stress^[^35–38^]^. Patient education is foundational and should encompass instruction on disease mechanisms, medication adherence, inhaler technique, and the development of personalized asthma action plans that help patients recognize worsening symptoms and initiate appropriate responses (Table 5)^[^39–41^]^.

Exacerbations represent acute deteriorations in asthma control that necessitate prompt intervention. The GINA framework for exacerbation management includes increased administration of inhaled SABA, the initiation or escalation of oral corticosteroids, and escalation of care for patients who do not respond to outpatient treatment. Hospitalization may be required in severe cases to provide intensive monitoring and therapy^[^42–44^]^. A central goal of asthma care is the prevention of such episodes through the consistent use of controller medications and avoidance of identified triggers. Asthma control is evaluated based on symptom burden, nocturnal awakenings, reliever use, and limitations in activity. GINA categorizes control status as well-controlled, partly controlled, or uncontrolled, guiding clinicians in adjusting therapy accordingly. Ongoing follow-up is crucial for assessing control, reinforcing self-management, and optimizing long-term outcomes^[^45–47^]^.

Special populations merit tailored considerations. Attention to growth and development, appropriate device selection, and caregiver education are critical in pediatric patients. Maintaining adequate asthma control during pregnancy is essential for preventing maternal and fetal complications. Among older adults, polypharmacy, comorbid conditions, and age-related changes in pharmacokinetics and lung physiology must be considered when selecting therapy^[^48,49^]^.

Recent updates to GINA guidelines have integrated emerging evidence and therapeutic innovations. The previous reliance on SABA monotherapy, even mild asthma, has been revised. The current recommendation favors the use of ICS-formoterol as both a maintenance and reliever therapy – a strategy known as single maintenance and reliever therapy. This approach offers consistent anti-inflammatory action and improved prevention of exacerbations, even in patients with infrequent symptoms^[^50–52^]^. The guidelines also emphasize personalized asthma management, recognizing the heterogeneous nature of the disease and the need to tailor interventions based on phenotype, biomarker profiles, comorbidities, and individual preferences. Personalized treatment strategies, especially in biologic agents, enable more precise targeting of underlying immunologic pathways and are particularly effective in managing eosinophilic or allergic asthma subtypes. GINA also recognizes the growing role of advanced diagnostic tools, such as fractional exhaled nitric oxide, in monitoring airway inflammation and guiding therapeutic decisions ^[^53–55^]^.

According to NHLBI guidelines, asthma diagnosis begins with a detailed clinical evaluation focused on classic symptoms – wheezing, shortness of breath, chest tightness, and cough – especially at night or early morning. These symptoms are often episodic and variable in severity. Spirometry is essential to confirm airflow limitation and reversibility. A post-bronchodilator increase in FEV1 of ≥12% supports a diagnosis of reversible airway obstruction^[^31–33^]^. Asthma treatment follows a stepwise approach based on symptom severity and control. Short-acting beta-agonists (SABAs) like albuterol are the first-line agents for quick symptom relief for intermittent asthma. SABAs provide rapid bronchodilation by stimulating β2-adrenergic receptors, reversing bronchospasm, and improving airflow^[^56–58^]^. For persistent asthma, daily long-term control therapy is recommended. ICS are the preferred first-line agents, as they effectively reduce airway inflammation and prevent exacerbations. Common ICS options include fluticasone, budesonide, and beclomethasone. Doses are titrated to the lowest effective amount needed to maintain control ^[^35^]^. If asthma remains uncontrolled with ICS alone, LABAs such as salmeterol or formoterol may be added. These provide sustained bronchodilation and are often delivered in fixed-dose combination inhalers alongside ICS for improved adherence and convenience^[^36^]^.

NHLBI recommends biologic therapies targeting specific inflammatory pathways for severe, treatment-resistant asthma. These include monoclonal antibodies like omalizumab (anti-IgE), mepolizumab, reslizumab, benralizumab (anti-IL-5/IL-5R), and dupilumab (anti-IL-4Rα). Administered subcutaneously or intravenously, these biologics have effectively reduced exacerbations, improved lung function, and enhanced quality of life in patients with eosinophilic or allergic asthma phenotypes ^[^37^]^.

NHLBI emphasizes the role of environmental control and lifestyle modifications in asthma management. Key strategies include avoiding known triggers such as allergens (e.g. dust mites, pet dander, pollen), irritants (e.g. tobacco smoke, pollution), respiratory infections, and strenuous exercise. To reduce exposure and improve outcomes, patients should implement control measures like using allergen-proof covers, maintaining indoor air quality, and avoiding smoking environments^[^38^]^. Education on correct inhaler technique, medication adherence, and early recognition of symptom worsening is vital. Regular follow-up visits are essential to reassess asthma control, monitor lung function, and adjust treatment as needed. Tools like the Asthma Control Test and Asthma Control Questionnaire are recommended to guide treatment decisions objectively. NHLBI also underscores the importance of patient empowerment and self-management skills to ensure long-term disease control^[^59–61^]^. International and regional clinical guidelines vary in their approach to asthma management, reflecting differences in healthcare systems, available resources, and patient populations. A comparative analysis reveals substantial variation in guideline scope, diagnostic tools, pharmacologic and non-pharmacologic strategies, and emphasis on research and special populations (Table 6). These differences underscore the importance of contextualizing guideline recommendations to the specific needs of patient populations and healthcare infrastructures.

**Table 6:** Comparison of international and regional asthma management guidelines

Guideline	Organization	Scope	Key features	Diagnostic tools	Treatment approach	Non-pharmacological recommendations	Special populations considered	Research and development emphasis
GINA Guidelines	Global Initiative for Asthma	Global	Stepwise approach, prevention focus	Spirometry, Peak Flow Meter	ICS, LABA, Biologics	Trigger avoidance, patient education	Children, pregnant women, elderly	High
NHLBI Guidelines	National Heart, Lung, and Blood Institute	US	Comprehensive, evidence-based	Spirometry, FeNO testing	ICS, LABA, LTRA	Allergen control, smoking cessation	Children, elderly	High
BTS/SIGN Guidelines	British Thoracic Society and Scottish Intercollegiate Guidelines Network	UK	Detailed, evidence-based	Spirometry, Peak Flow Meter	ICS, LABA, LTRA	Allergen avoidance, smoking cessation	Children, pregnant women, elderly	High
Australian Asthma Handbook	National Asthma Council Australia	Australia	Practical, comprehensive	Spirometry, Peak Flow Meter	ICS, LABA, LTRA	Allergen management, lifestyle advice	Children, pregnant women, elderly	Medium
CTS Asthma Guidelines	Canadian Thoracic Society	Canada	Personalized, comprehensive	Spirometry, Peak Flow Meter	ICS, LABA, Biologics	Trigger management, patient education	Children, pregnant women, elderly	High
WHO Guidelines	World Health Organization	Global	Public health focus	Spirometry, Peak Flow Meter	ICS, LABA, SABA for relief	Environmental control, smoking cessation	Vulnerable populations, rural areas	Medium
ATS/ERS Guidelines	American Thoracic Society and European Respiratory Society	US and Europe	Detailed, research-based	Spirometry, Bronchoprovocation	ICS, LABA, Biologics	Environmental control, patient education	Children, occupational asthma	High
CHEST Guidelines	American College of Chest Physicians	US	Evidence-based, clinical focus	Spirometry, Peak Flow Meter	ICS, LABA, LAMA	Allergen management, smoking cessation	Children, elderly	Medium
NICE Asthma Guidelines	National Institute for Health and Care Excellence	UK	Practical, patient-centered	Spirometry, FeNO testing	ICS, LABA, LTRA	Environmental control, education	Children, pregnant women, elderly	High
ERS/EAACI Guidelines	European Respiratory Society and European Academy of Allergy and Clinical Immunology	Europe	Comprehensive, research-based	Spirometry, Peak Flow Meter	ICS, LABA, Biologics	Allergen management, patient education	Children, occupational asthma	High
GAN Asthma Guidelines	Global Asthma Network	Global	Public health focus	Spirometry, Peak Flow Meter	ICS, LABA	Environmental control, smoking cessation	Vulnerable populations, rural areas	Medium
APAAACI Asthma Guidelines	Asia Pacific Association of Allergy, Asthma, and Clinical Immunology	Asia-Pacific	Regional focus, practical	Spirometry, Peak Flow Meter	ICS, LABA, LTRA	Allergen management, lifestyle advice	Children, elderly, rural areas	Medium
ALAT Asthma Guidelines	Latin American Thoracic Association	Latin America	Regional focus, evidence-based	Spirometry, Peak Flow Meter	ICS, LABA, LTRA	Environmental control, patient education	Children, pregnant women	Medium
ALDT Asthma Guidelines	African Union of Lung Disease and Tuberculosis	Africa	Context-specific, comprehensive	Spirometry, Peak Flow Meter	ICS, LABA, LTRA	Allergen management, smoking cessation	Children, pregnant women, rural areas	Medium
CNRC Asthma Recommendations	Canadian Network for Respiratory Care	Canada	Detailed, patient-focused	Spirometry, Peak Flow Meter	ICS, LABA, Biologics	Trigger management, patient education	Children, pregnant women, elderly	High

Source: Authors’ creations. International and regional asthma guidelines differ in scope, diagnostic criteria, treatment strategies, and emphasis on research, reflecting diverse healthcare priorities and patient populations. Guidelines like GINA, NHLBI, BTS/SIGN, and WHO offer varied approaches shaped by clinical evidence, public health focus, and resource availability, with specific considerations for vulnerable groups and evolving research landscapes.

## Other information

Our review was not registered, and a formal protocol was not prepared; therefore, no amendments were made. The authors received no financial or non-financial support, and no funders or sponsors had any role in the review. The authors declare no competing interests, and all data, materials, and relevant documentation used in this review are included in the published article.

## Concluding remarks

Our systematic review highlights the central role of ICS and the growing importance of biologics in the personalized treatment of asthma, particularly for patients with moderate to severe disease. Critical gaps remain while the evidence base has expanded considerably in recent years, especially regarding targeted therapies and digital health innovations. These include limited data on long-term outcomes, a lack of head-to-head comparisons between biologics, and the underrepresentation of specific populations such as children, older adults, and pregnant individuals.

## Call to action

To advance asthma care, we call on clinicians, researchers, and policymakers to act collaboratively. Clinicians should adopt evidence-based, phenotype-driven strategies that prioritize individualized patient needs. Researchers should focus on generating high-quality, inclusive studies that assess the long-term effectiveness and comparative value of existing and emerging therapies. Policymakers must support equitable access to proven interventions and ensure clinical guidelines are effectively implemented in primary care settings.

Only through such coordinated efforts can we close persistent gaps in asthma care, reduce the global burden of disease, and ensure that all patients – regardless of age, socioeconomic status, or geographic location – receive optimal, evidence-based management.

## Data Availability

This published article and its supplementary information files include all data generated or analyzed during this study.
